# Reply to Keles et al. Comment on “Dalboni da Rocha et al. Artificial Intelligence for Neuroimaging in Pediatric Cancer. *Cancers* 2025, *17*, 622”

**DOI:** 10.3390/cancers17111778

**Published:** 2025-05-26

**Authors:** Josue Luiz Dalboni da Rocha, Jesyin Lai, Pankaj Pandey, Phyu Sin M. Myat, Zachary Loschinskey, Asim K. Bag, Ranganatha Sitaram

**Affiliations:** 1 Department of Radiology, St. Jude Children’s Research Hospital, Memphis, TN 38105, USA; jesyin.lai@stjude.org (J.L.); pankaj.pandey@stjude.org (P.P.); phyu.sin.m.myat@vanderbilt.edu (P.S.M.M.); zfl@bu.edu (Z.L.); asim.bag@stjude.org (A.K.B.); 2 Department of Chemical and Biomedical Engineering, University of Missouri-Columbia, Columbia, MO 65211, USA; 3 Department of Biomedical Engineering, Boston University, Boston, MA 02215, USA

We thank the readers for their thoughtful reading of our review, “Artificial Intelligence for Neuroimaging in Pediatric Cancer”, and for their interest in discussing aspects of segmentation performance metrics [1].

Our intention was to provide a concise and accessible overview of key developments in this rapidly evolving field. To enhance readability, we summarized some of the most relevant recent approaches in a single table. As noted by the commenters, performance metrics across studies may differ in scope, measurement types, and validation strategies. We appreciate the opportunity to clarify that the values in Table 1 were intended as representative highlights—a simplified snapshot of recent work—rather than for direct cross-study comparison.

These distinctions—such as those between whole-tumor and subregion segmentation, metric types, or validation cohorts—reflect the range of methodological approaches found in the literature. We appreciate the readers’ attention to these aspects and agree that interpretive comparisons by a general audience require careful consideration of each study’s specific context. We will take these suggestions into account in future publications, where a more comprehensive and multi-dimensional presentation of results can be provided.

Nevertheless, the observations raised do not affect the overall conclusions of our review, which emphasized the promising role of AI-based neuroimaging tools in pediatric oncology. We appreciate the constructive nature of this exchange.
